# Effects of phenylcapsaicin on aerobic capacity and physiological parameters in active young males: a randomized, triple-blinded, placebo-controlled, crossover trial

**DOI:** 10.3389/fphys.2023.1190345

**Published:** 2023-05-09

**Authors:** Pablo Jiménez-Martínez, Carlos Alix-Fages, Danica Janicijevic, Sergio Miras-Moreno, Sara Chacón-Ventura, Juan J. Martín-Olmedo, Juan Carlos De La Cruz-Márquez, Francisco J. Osuna-Prieto, Lucas Jurado-Fasoli, Francisco J. Amaro-Gahete, Amador García-Ramos, Juan C. Colado

**Affiliations:** ^1^ Research Group in Prevention and Health in Exercise and Sport (PHES), University of Valencia, Valencia, Spain; ^2^ Life Pro Nutrition Research Center, INDIEX, Madrid, Spain; ^3^ ICEN Institute, Madrid, Spain; ^4^ Applied Biomechanics and Sport Technology Research Group, Autonomous University of Madrid, Madrid, Spain; ^5^ Research Academy of Human Biomechanics, The Affiliated Hospital of Medical School of Ningbo University, Ningbo University, Ningbo, China; ^6^ Faculty of Sports Science, Ningbo University, Ningbo, China; ^7^ Department of Physical Education and Sport, Faculty of Sport Sciences, University of Granada, Granada, Spain; ^8^ Department of Physical and Sports Education, Faculty of Sport Sciences, Sport and Health University Research Institute (iMUDS), University of Granada, Granada, Spain; ^9^ Research Institute in Health Pere Virgili, University Hospital of Tarragona Joan XXIII, Tarragona, Spain; ^10^ Department of Sports Sciences and Physical Conditioning, Universidad Católica de la Santísima Concepción, Concepción, Chile

**Keywords:** substrate oxidation, ergogenic aid, phenylcapsaicin, exercise metabolism, exercise capacity

## Abstract

**Objective:** Phenylcapsaicin (PC) is a new capsaicin analog which has exhibited a higher bioavailability. This sudy assessed the effects of a low dose (LD) of 0.625 mg and a high dose (HD) of 2.5 mg of PC on aerobic capacity, substrate oxidation, energy metabolism and exercise physiological variables in young males.

**Materials and methods:** Seventeen active males (age = 24.7 ± 6.0 years) enrolled to this randomized, triple-blinded, placebo-controlled, crossover trial. Participants attended the laboratory on 4 sessions separated by 72–96 h. A submaximal exercise test [to determine maximal fat oxidation (MFO) and the intensity at MFO (FATmax)] followed by a maximal incremental test (to determine VO2_max_) were performed in a preliminary session. The subsequent sessions only differed in the supplement ingested [LD, HD or placebo (PLA)] and consisted of a steady-state test (60 min at FATmax) followed by a maximal incremental test. Energy metabolism, substrate oxidation, heart rate, general (gRPE) and quadriceps (RPEquad) rate of perceived exertion, skin temperature and thermal perception were tested.

**Results:** Clavicle thermal perception was lower in HD compared to PLA and LD (*p* = 0.04) across time. HD reduced maximum heart rate in comparison to PLA and LD (*p* = 0.03). LD reported higher general RPE (RPEg) values during the steady-state test compared to PLA and HD across time (*p* = 0.02). HD and LD elicited higher peak of fat oxidation during the steady-state test compared with PLA (*p* = 0.05). Intra-test analyses revealed significant differences for fat oxidation (FATox) in favor of HD and LD compared to PLA (*p =* 0.002 and 0.002, respectively), and for carbohydrate oxidation (CHOox) (*p =* 0.05) and respiratory exchange ratio (RER) (*p =* 0.03) for PLA. In the incremental test, only general RPE at 60% of the maximal intensity (W) differed favoring HD (*p* ≤ 0.05).

**Conclusion:** Therefore, PC may contribute to increase aerobic capacity through the improvement of fat oxidation, maximum heart rate and perceptual responses during exercise.

## 1 Introduction

Capsaicinoids are a group of substances found in spicy fruits such as chili peppers (González-Cano et al., 2022; Jiménez-Martínez et al., 2022). Among the variety of substances included in this category, capsaicin has emerged as the most used ergogenic compound (de Moura e Silva et al., 2021). Capsaicin vanillyl moiety is responsible for its pungent properties, which are associated with its ergogenic effects (Jiménez-Martínez et al., 2022). However, although capsaicin spiciness has been proposed as an athletic enhancer mechanism, new non-pungent analogs have emerged as a plausible alternative (Luo et al., 2011). Capsinoids are a group of capsaicin non-pungent analogs (e.g., capsiate) found in sweet peppers (Lang et al., 2009). Although capsaicinoids and capsinoids have a similar chemical structure, these substances present differences in their functional properties and their metabolism. Accordingly, capsinoids are rapidly metabolized and conjugated after their ingestion (EFSA, 2012), resulting in non-detectable circulating levels in the bloodstream (EFSA, 2012). By contrast, after their ingestion, different capsaicin formulations have a fast effect on different tissues, such as the small intestine, liver, and stomach (Turck et al., 2019). Recent advances in this field have discovered a new capsaicin analog called phenylcapsaicin (PC), which has shown a higher bioavailability (Turck et al., 2019) in comparison to purified capsaicin. PC formulations are composed of 98% PC and 1.0%–1.5% of cellulose and lipidic excipients as vehicles. As a consequence, PC might reduce the ergogenic dose of capsaicin (Turck et al., 2019) and consequently the spicy perception and gastrointestinal discomfort after its ingestion.

The ergogenic effects of capsaicinoids and capsinoids are mediated by their interaction with the transient receptor vanilloid 1 (TRPV1) (Jiménez-Martínez et al., 2022). TRPV1 receptors are found in afferent III and IV nerve fibers, which are key for the development of central fatigue during exercise (Collins et al., 2018) as the increased firing in these afferents could inhibit motor neurons firing directly and through the presynaptic inhibition of Ia afferents reducing or affecting supraspinal levels to reduce the descending drive to the motor units (Taylor et al., 2016; Alix-Fages et al., 2022a). Besides, ingesting these substances enhance muscle contraction as a consequence of the improvement in motoneurons recruitment, calcium release, and perceived analgesia (de Moura e Silva et al., 2021). Additionally, other potential ergogenic mechanisms related to TRPV1 stimulation concern metabolic effects such as an increase in fatty acid oxidation (more FFAs available for beta-oxidation), an increase in glycogen sparing, and a positive effect in the acetylcholine turnover (de Moura e Silva et al., 2021). However, current human evidence regarding the positive metabolic impact of these substances is scarce, albeit the plausible mechanisms aforementioned are well documented in preclinical models (Basith et al., 2016; Jiménez-Martínez et al., 2022).

In the present study, only active males were recruited as previous research has exhibited that the effects of capsaicin on substrate oxidation are unaffected by the sex of the subjects (Lejeune et al., 2003). To date, only two studies have reported the metabolic effects of a capsaicinoid on substrate oxidation (Lim et al., 1997; Rossi et al., 2022). In the first study, participants exhibited equal RER values after 12 mg of capsiate supplementation during exercise performed at 70% of the maximal aerobic speed (Rossi et al., 2022). In the second study, a meal with 10 g of hot peppers 2.5 h prior to 1 h of aerobic exercise at 60% VO2_peak_ showed an increase in RER compared to placebo (Lim et al., 1997). An important limitation in current research is that human substrate oxidation is not usually reported in previous studies (Santos et al., 2022). For instance, 10 mg/kg of capsiate supplementation 60 min prior to aerobic exercise decreased RER in mice males (Santos et al., 2022). On the other hand, 12 mg of dihydrocapsiate supplementation has not exhibited significant differences in fat oxidation (FATox), non-esterified fatty acids, energy expenditure (EE), and skin temperature in sedentary overweight men during aerobic exercise (Osuna-Prieto et al., 2022). Furthermore, most current studies do not standardize maximal fat oxidation (MFO) measurement in the pre-test, which may bias the metabolic endpoints measured (Santos et al., 2022). Concerning the metabolic effects of these substances on high-intensity training (i.e., resistance training and high-intensity interval training), 12 mg of capsaicin supplementation has exhibited lower lactate levels compared to placebo after four sets until failure in the squat exercise (de Freitas et al., 2018a). This reduction in lactate values has also been reported in other high-intensity tasks, such as repeated sprints after 12 mg of capsaicin supplementation (de Freitas et al., 2018b). Aditionally, previous research has documented a negligible effect of capsaicin supplementation on heart rate (Giuriato et al., 2022). Overall, evidence regarding the metabolic impact of capsaicinoids and capsinoids during exercise is scarce and inconclusive. Furthermore, the effects of PC on physiological exercise variables and sport performance have not been tested yet. For this reason, if new capsaicin formulations, which are supposed to be more bioavailable (e.g., PC), produce an ergogenic effect on substrate oxidation, energy metabolism and aerobic capacity and if a dose threshold exists have to be investigated. Note that PC intake should reduce spicy perception and gastrointestinal discomfort, as the ergogenic dose might be lower compared to capsaicin (Turck et al., 2019).

Therefore, this study aimed to assess the effects of a low dose (LD) of 0.625 mg and a high dose (HD) of 2.5 mg of PC on aerobic capacity, energy metabolism, substrate oxidation and other physiological variables such as circulating lactate levels, energy expenditure, body temperature response, ratings of perceived exertion (RPE) and perceived temperature compared to placebo in active males. We hypothesized that PC supplementation will increase FATox, skin body temperature, mechanical performance and energy expenditure while will decrease RPE in a dose-dependent manner.

## 2 Materials and methods

### 2.1 Participants

Sample size calculation was performed using the G* POWER software (Heinrich-Heine-Universität Düsseldorf, Germany) with an alpha of 0.05, an effect size of 0.4 and a statistical power of 0.80. Based on previous studies (Josse et al., 2010; Osuna-Prieto et al., 2022), 12 participants were required to establish statistical differences between conditions. To ensure the detection of differences 17 physically active males were enrolled in the study (Table 1). Participants enrolled in the study through a poster that was shared on social media. None of the participants reported any physical limitation or health condition that could compromise cycling performance. Participants were instructed not to perform any intense physical exercise during the 2 days preceding each visit to the laboratory and from consuming stimulant beverages or any dietary supplement within 24 h preceding each testing session. Before being included in the study, all potential participants were comprehensively informed about the study purpose, procedures and the benefits, risks, and discomforts that might result from participation. Each participant provided informed consent and was free to withdraw from the study at any time. The study protocol adhered to the tenets of the last revised Declaration of Helsinki and was approved by the Institutional Review Board (blinded for peer review).

**TABLE 1:** Characteristics of the study participants (*n* = 17).

	Mean ± standard deviation
Age (years)	24.7 ± 6.0
Height (cm)	175.9 ± 7.6
Body mass (kg)	77.2 ± 11.7
Muscle mass (%)	41.2 ± 6.2
Fat mass (%)	6.9 ± 5.3
FATmax (W)	68.8 ± 37.7
MFO (g/min)	0.27 ± 0.09

FATmax, intensity linked to maximal fat oxidation; MFO, maximal fat oxidation.

### 2.2 Experimental design

**FIGURE 1:**
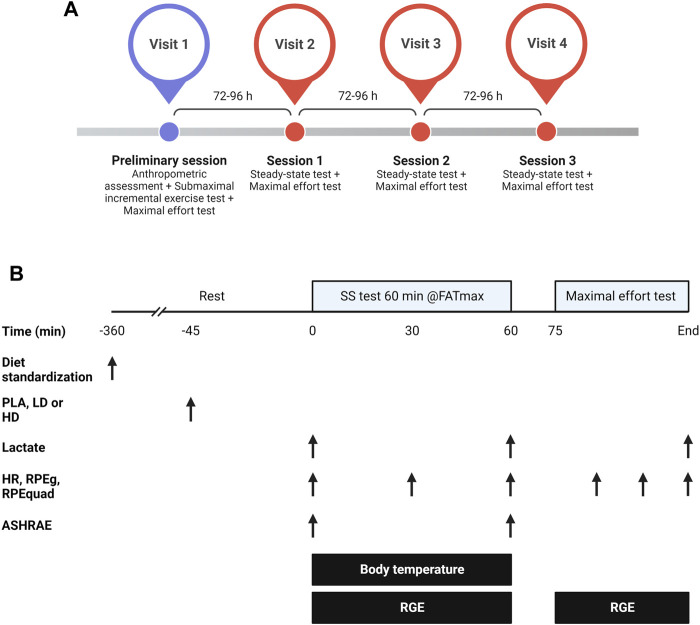
General overview of the study **(A)** and design of the experimental sessions **(B)** aiming to analyze the effects of two doses of phenylcapsaicin on metabolic, thermal, perceptual, and mechanical responses to steady-state and maximal cycling tests. HD, high dose; LD, low dose; PLA, placebo; HR, heart rate; RGE, respiratory gas exchange; RPEg, general ratings of perceived exertion; RPEquad, ratings of perceived exertion of quadriceps; SS, steady-state. Created with BioRender.com.

A randomized, triple-blinded, placebo-controlled crossover trial was used to analyze the effects of PC on energy expenditure and substrate oxidation, skin body temperature, heart rate and perceptual responses to submaximal steady-state and maximal effort cycling tests (Figure 1). Participants attended the laboratory four times, separated by 72–96 h to ensure a complete recovery from central and peripheral fatigue (Carroll et al., 2017; Alix-fages et al., 2023). Before the preliminary session, participants anthropometrical [(i.e., height, body mass, % muscle, % body fat (Tanita BC 418 segmental, Tokyo, Japan; Seca 202 Stadiometer, Seca Ltd., Hamburg, Germany)] and sociodemographic characteristics were obtained (see Table 1). Then, two tests, a submaximal incremental exercise test followed by a maximal effort incremental test, were conducted in the preliminary session. The submaximal exercise test was used to determine the MFO and the cycling power values (W) at MFO (FATmax intensity). The maximal effort test assessed maximal oxygen consumption (VO2_max_) and the maximal cycling power achieved during the test. The three experimental sessions were identical, only differing in the supplement (PLA, LD of PC, and HD of PC), which was only administered 45 min before the first cycling task. In each experimental session, participants performed a steady-state test (60 min at FATmax) followed by a maximal incremental effort test (25 W increments every min until volitional exhaustion). Each participant was constantly tested at the same time of the day and under similar environmental conditions (22ºC–24°C and 55% humidity).

### 2.3 Supplementation procedures

Capsules were ingested with water. The contents of the capsules were as follows: a low dose (LD) of 0.625 mg of PC, a high dose (HD) of 2.5 mg HD of PC (Axivite, Malmö, Sweden), and a PLA composed of maltodextrin and excipients. According to EFSA, both PC doses are considered in the safety range proposed by its expert panel judgment (Turck et al., 2019). Supplements and placebo were encapsulated and packaged with alphanumeric labels to ensure blinding. Accordingly, an independent technician (i.e., not involved in the study) prepared the capsules in the original producer’s industry (Life Pro Nutrition industries, Madrid, Spain). Participants selected one capsule of the daily randomized assigned condition under the supervision of at least one researcher. To reduce possible bias, an independent researcher assigned participants to each condition with the Research Randomizer online software (www.randomizer.org). Packages and capsules were indistinguishable in appearance, smell and taste and their content was only revealed after an independent researcher performed the statistical analyses to prevent evaluators’ bias. Adverse effects were not reported for any of the conditions.

### 2.4 Dietary intake standardization

To ensure intra and inter-individual reliability in metabolic variables (e.g., FATox), dietary intake was standardized at least 6 h previous to exercise testing (Amaro-Gahete et al., 2018; Rothschild et al., 2022). For this purpose, participants performed each test with at least 6 h of fasting prior to the start of each session. The last meal before the fasting period was standardized with 45 g of maltodextrin powder and 30 g of protein powder for all participants (Life Pro Nutrition industries, Madrid, Spain).

### 2.5 Exercise procedures

The three tests were performed employing an electronically braked cycle ergometer (Excalibur Sport; Lode, Groningen, Netherlands). Respiratory gas exchange was monitored during all tests with a gas analyzer (Ergocard CPX, Medisoft, Belgium). The testing procedures and variables collected in each test are described below:

#### 2.5.1 Submaximal incremental exercise test

This test was used to determine MFO and FATmax in the preliminary session. The exercise protocol started with a 3 min stage at 25 W, and the intensity was increased in steps of 25 W every 3 min until the respiratory exchange ratio (RER) was ≥1 for at least 30 s (Sanchez-Delgado et al., 2018). During the submaximal exercise test, VO2 and carbon dioxide production (VCO2) data were averaged over the last 60 s of each 3 min stage. Then, FATox was calculated from the aforementioned values. FATox values (g/min) from the different stages of the submaximal exercise test were plotted against the relative exercise intensity (W). Third-degree polynomial regression was built to determine the absolute MFO (g/min) (Midgley et al., 2007; Sanchez-Delgado et al., 2018). EE was estimated with Weir’s abbreviated equations (Weir, 1949) and FATox and CHOox with Frayn’s stoichiometric equations assuming a negligible urinary nitrogen excretion (Frayn, 2016; Osuna-Prieto et al., 2022). Subsequently, RER was calculated from the CHOox and FATox obtained data. This procedure was further used for the next sessions. The equations used are presented hereunder:

$$EE\;\left(kcal/\min\right)=\left(1.106\;*\;{VCO}_{2}\right)+\left(3.941\;*\;{VO}_{2}\right)$$

$$RER=\left({VCO}_{2}/{VO}_{2}\right)$$

$$CHOox\;\left(g/\min\right)=\left(4.55\;*\;{VCO}_{2}\right)-\left(3.21\;*\;{VO}_{2}\right)$$

$$FATox\;\left(g/\min\right)=\left(1.67\;*\;{VCO}_{2}\right)-\left(1.67\;*\;{VO}_{2}\right)$$

#### 2.5.2 Maximal effort test

This test was performed in the preliminary and the three experimental sessions, 15 min after the first test of each session. The exercise protocol started with a 1 min stage at 25 W, and the intensity was increased in steps of 25 W every min until i) volitional exhaustion was reached, or ii) participants had to stop because of peripheral fatigue. The dependent variables considered in this test were: i) final stage completed in the protocol, ii) VO2_max_, iii) circulating lactate concentration recorded 90 s after test cessation with a portable lactate analyzer (Lactate PRO2, Arkray, Kyoto, Japan) (Crotty et al., 2021), iv) general (RPEg) and quadriceps (RPEquad) ratings of perceived exertion (RPE 0-10) and heart rate (Polar RS800, Polar Electro Inc., Woodbury, NY, United States) (Hernando et al., 2018) at an intensity representing the 30%, 60%, and 90% of the maximal power attained at the final stage completed in the preliminary session. Lactate blood was extracted with a sterilized lancet after cleaning and drying the fingertip of participants before each attempt. VO2 was monitored using a galvanic fuel cell, and VCO2 was evaluated using a non-dispersive infrared sensor. The gas analyzer was calibrated following standard gas concentrations as suggested by the manufacturer.

#### 2.5.3 Steady-state test

Participants cycled for 60 min at the intensity of FATmax determined in the preliminary session. Circulating lactate, RPEg, RPEquad, and heart rate were recorded pre-exercise, in the middle of the test (30 min), and at the end (60 min). The skin body temperature was also recorded throughout the test with a set of 8 DS-1922 L iButtonTM wireless thermometers (Thermochron, Dallas, TX, United States) (van Marken Lichtenbelt et al., 2006; Smith et al., 2010) attached to the participant’s skin in different places: i) forehead, ii) right scapula, iii) left chest, iv) right% deltoid), v) left elbow, vi) left hand, vii) right thigh and viii) right gastrocnemius. Data were processed as mean blocks of 5 min. Consequently, a total of 12 temperature stages were recorded and averaged for the final analysis.

The equation used to assess the effects of PC and PLA on body temperature is described hereunder:

$$\begin{array}{ll} Overall\;mean\;skin\;temperature & =\left(Forehead*0.07\right)+\left(Right\;Scapula*0.175\right)+\left(Left\;Chest*0.175\right)+\left(Right\;Deltoid*0.07\right)\\ & +\left(Left\;Elbow*0.07\right)+\left(Left\;Hand*0.05\right)+\left(Right\;Thigh*0.19\right)+\left(Right\;Gastrocnemius*0.2\right) \end{array}$$

Martinez-Tellez et al. (2017) the mean and maximum heart rates recorded throughout the test were also compared between the experimental conditions. The substrate oxidation (fat and carbohydrates), energy expenditure, and RER during the 60 min steady-state test were also estimated and compared between the conditions. FATox, CHOox and EE were also expressed as the area under the curve (AUC) using the trapezoidal rule. For metabolic variables, the highest value achieved during the test (i.e., peak) and the individual analysis of each stage (i.e., intra-test analysis) were performed. Finally, the American Society of Heating, Refrigerating, and Air Conditioning Engineers (ASHRAE) scale was used to record thermal perception before and after the test. The scale was recorded for the following body areas: i) clavicle, ii) abdominal, iii) arms, iv) hands, v) legs, vi) feet, and vii) overall body. Each item is scored with the following values: cold (−3), cool (−2), slightly cool (−1), neutral (0), slightly warm (1), warm (2), to hot (3).

### 2.6 Statistical analysis

Data are presented as means and standard deviations (Mean ± SD). The normal distribution of all the variables presented was tested with the Shapiro-Wilk test, and the homogeneity of the variances with the Levene’s test (*p* > 0.05). A two-way repeated measures analysis of variance (ANOVA) (condition × time) was used to analyze the effect of the supplementation (LD, HD, PLA) across the time on each dependent metabolic, performance, and perceptual variable. F value was retrieved from each ANOVA calculation. A Bonferroni *post hoc* comparison was performed when ANOVA significance was reached. A one-way repeated measures ANOVA was used to compare intra-test effects for each stage and for the maximal metabolic values of the steady-state test. The Greenhouse-Geisser correction was applied when Mauchly’s sphericity test was significant (*p* ≤ 0.05). For non-parametric data, Friedman’s test and *post hoc* Wilcoxon corrections were used instead. Statistical analyses were performed using the software package SPSS (IBM SPSS version 25.0, Chicago, IL, United States). Statistical significance was set at *p* ≤ 0.05. The magnitude of the differences was assessed with partial eta squared values (ƞp^2^) derived from ANOVAs and were interpreted as low (<0.04), moderate (0.04–0.13) and large (>0.13) for all the parametric outcomes. The effect size of the *post hoc* comparisons was calculated by means of Cohen’s d, which was interpreted as a low (<0.50), moderate (0.50–0.80), or large effect (>0.80) (Cohen, 1988).

## 3 Results

### 3.1 Circulating lactate levels, RPE and heart rate during steady-state test

Two-way repeated measures ANOVAs did not reveal significant differences for condition in circulating lactate levels, general and local RPE, nor heart rate (*p* range = 0.08–0.56) (Table 2). Significant differences were reported in time for heart rate, RPEg, and RPEquad (*p* ≤ 0.001) (Table 2). Bonferroni *post hoc* analyses revealed significant differences for all time comparisons (pre, “30” and “60”) in heart rate, RPEg, and RPEquad (*p* range <0.001–0.004). Significant condition × time interaction was only found for RPEg due to higher values in LD compared to PLA (d = 27) and HD (d = 0.27) (Table 2).

**TABLE 2:** Two-way repeated measures analysis of variance (ANOVA) comparing circulating lactate levels, general and local RPE, and heart rate between the different experimental conditions during the steady-state test.

Variable	Time	Condition			ANOVA		
		PLA	LD	HD	Condition	Time	Condition × time
Lactate (mmol/L)	Pre	1.54 ± 0.38	2.08 ± 2.21	2.43 ± 3.49	F = 0.58, *p* = 0.56 ƞp^2^ = 0.04	F = 0.03, *p* = 0.86 ƞp^2^ = 0.02	F = 2.68, *p* = 0.08 ƞp^2^ = 0.16
	Post	3.37 ± 4.54	1.36 ± 0.43	1.63 ± 0.51			
RPEg (a.u.)	Pre	0.19 ± 0.40	0.06 ± 0.25	0.00 ± 0.00	F = 1.08, *p* = 0.33 ƞp^2^ = 0.06	F = 47.40, *p* < 0.001* ƞp^2^ = 0.76	F = 3.15, *p* = 0.02* ƞp^2^ = 0.17
	30′	1.94 ± 1.12	2.06 ± 1.53	2.06 ± 1.34			
	60′	2.75 ± 1.48	3.63 ± 2.31	2.94 ± 1.88			
RPEquad (a.u.)	Pre	0.25 ± 0.58	0.25 ± 0.45	0.06 ± 0.25	F = 2.65, *p* = 0.08 ƞp^2^ = 0.15	F = 44.30, *p* < 0.001* ƞp^2^ = 0.74	F = 1.62, *p* = 0.20 ƞp^2^ = 0.10
	30′	1.94 ± 1.29	2.56 ± 1.71	2.19 ± 1.56			
	60′	3.06 ± 1.61	3.69 ± 2.18	3.19 ± 1.80			
Heart rate (b/min)	Pre	70.4 ± 10.2	66.6 ± 8.6	67.9 ± 9.6	F = 2.28, *p* = 0.11 ƞp^2^ = 0.13	F = 64.86, *p* < 0.001* ƞp^2^ = 0.81	F = 0.85, *p* = 0.45 ƞp^2^ = 0.05
	30′	105.5 ± 15.9	103.1 ± 16.59	102.2 ± 17.8			
	60′	109.3 ± 19.6	107.4 ± 18.1	103.9 ± 17.4			

Mean ± standard deviation. PLA, Placebo; HD, high dose; LD, low dose; Pre; Before the start of the session; 30′, at minute 30 of steady-state test; 60′, at minute 60 of steady test; RPEg, general ratings of perceived effort; RPEquad, ratings of perceived effort in quadriceps; a.u., arbitrary units. * Significant difference: *p* ≤ 0.05.

### 3.2 *Skin body temperature*, maximal metabolic respiratory variables and mean and maximum heart rate during steady-state test

One-way repeated measures ANOVAs did not reveal significant differences in skin body temperature (*p* = 0.27) or mean heart rate (*p* = 0.24) (Table 3). Maximal carbohydrate oxidation, energy expenditure and RER did not differ between conditions (*p* ranged from 0.10 to 0.77). However, significant differences were found for maximum heart rate (*p* = 0.03) and MFO (*p* = 0.05) (Table 3), where PLA reached the maximum and HD the lowest values. However, Bonferroni *post hoc* did not revealed differences between conditions in any of the outcomes (*p* range = 0.09 to 0.99; d range = 0.20–0.31).

**TABLE 3:** One-way repeated measures analysis of variance (ANOVA) comparing skin body temperature, mean and maximum heart rate, and maximal metabolic respiratory variables between the three experimental conditions during the steady-state test.

Variable	Conditions			ANOVA
	PLA	LD	HD	
Skin body temperature (°C)	30.27 ± 1.02	30.48 ± 1.22	30.02 ± 1.22	F = 1.32, *p* = 0.27, ƞp^2^ = 0.09
Mean heart rate (bpm)	100.73 ± 14.96	99.97 ± 14.18	97.66 ± 15.22	F = 1.47, *p* = 0.24, ƞp^2^ = 0.09
Maximum heart rate (bpm)	114.13 ± 18.24	112.31 ± 18.53	108.75 ± 16.83	F = 3.23, *p* = 0.03*, ƞp^2^ = 0.19
FOpeak (g/min)	0.28 ± 0.09	0.32 ± 0.11	0.33 ± 0.11	F = 3.20, *p* = 0.05*, ƞp^2^ = 0.16
CHOOXpeak (g/min)	1.12 ± 0.60	1.03 ± 0.50	1.07 ± 0.50	F = 3.05, *p* = 0.10, ƞp^2^ = 0.15
MEEpeak (kcal/min)	5.99 ± 1.90	6.03 ± 1.85	6.10 ± 1.92	F = 0.20, *p* = 0.77, ƞp^2^ = 0.01
MRERpeak (g/min)	0.92 ± 0.05	0.91 ± 0.05	0.91 ± 0.06	F = 0.80, *p* = 0.45, ƞp^2^ = 0.05

Mean ± standard deviation. PLA, Placebo; HD, high dose; LD, low dose; FOpeak, Peak of fat oxidation; CHOOXpeak, Peak of carbohydrate oxidation; MEEpeak, Peak of energy expenditure; MRERpeak, Peak of respiratory exchange ratio. * Significant difference: *p* ≤ 0.05.

### 3.3 Thermal perception during steady-state test

**FIGURE 2:**
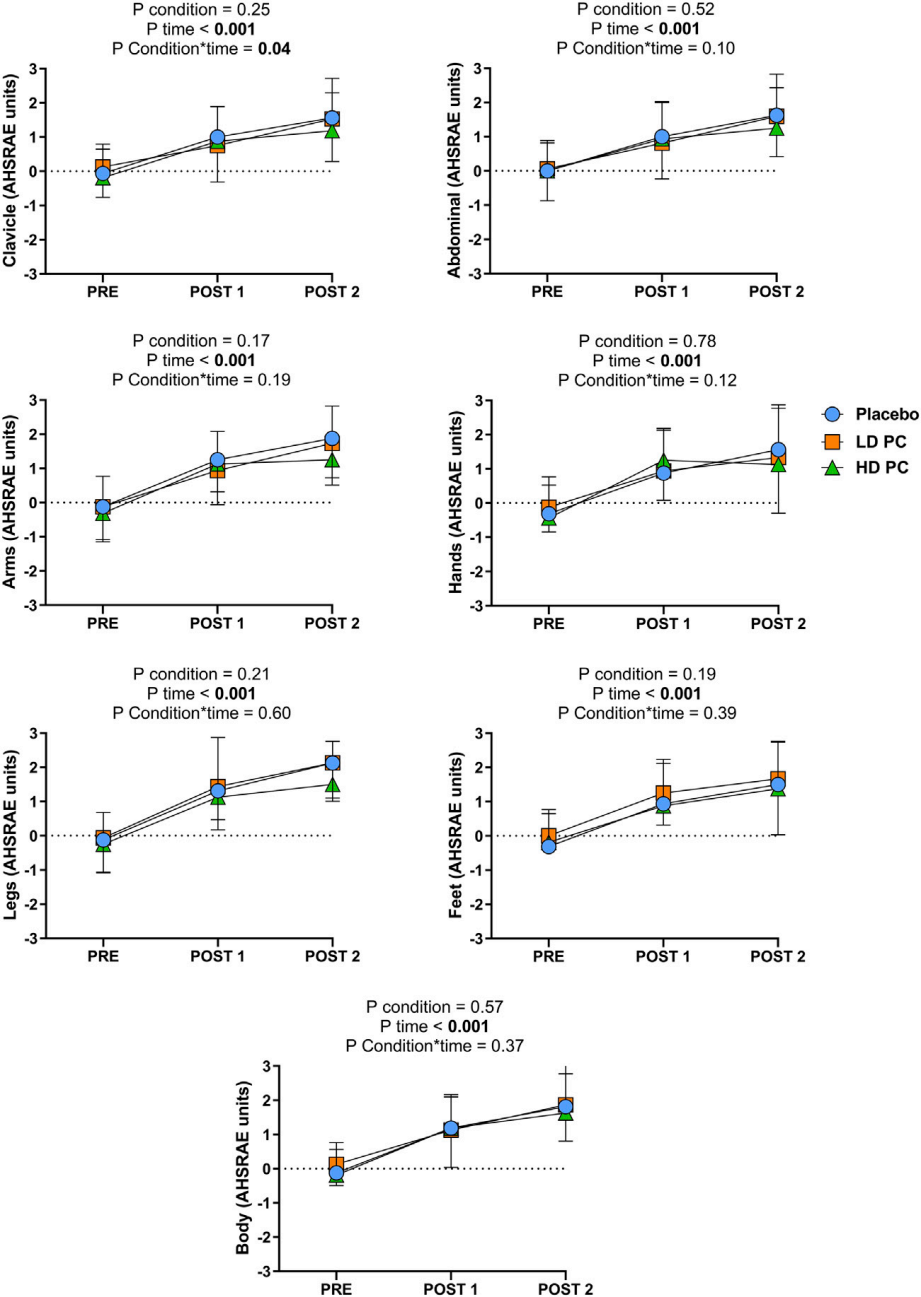
Two-way repeated measures analysis of variance (ANOVA) comparing the effects of consuming different dosis of phenylcapsaicin (HD and LD) or placebo (PLA) on thermal perception (ASHRAE scale) at different time points. PLA, Placebo; HD, High dose; LD, Low dose; PC, Phenylcapsaicin. PRE: Before the start of the session; Post 1: Between the steady state test and maximal effort test; Post 2: After the maximal effort test.

Significant differences for condition were not reported for any of the ASHRAE outcomes measured (*p* range = 0.17 to 0.78; ƞp^2^ range = 0.01–0.12). However, significant differences were reported in time for all the variables (*p* ≤ 0.001; ƞp^2^ range = 0.52–0.73). A significant condition × time interaction was found for the clavicle (*p* = 0.04; ƞp^2^ = 0.16) area due to the lower value of HD in comparison to LD an PLA. The other areas did not exhibit condition × time interactions (*p* range = 0.12 to 0.60; ƞp^2^ range = 0.04–0.12) (Figure 2).

### 3.4 Energy expenditure and substrate oxidation during steady-state test

**FIGURE 3:**
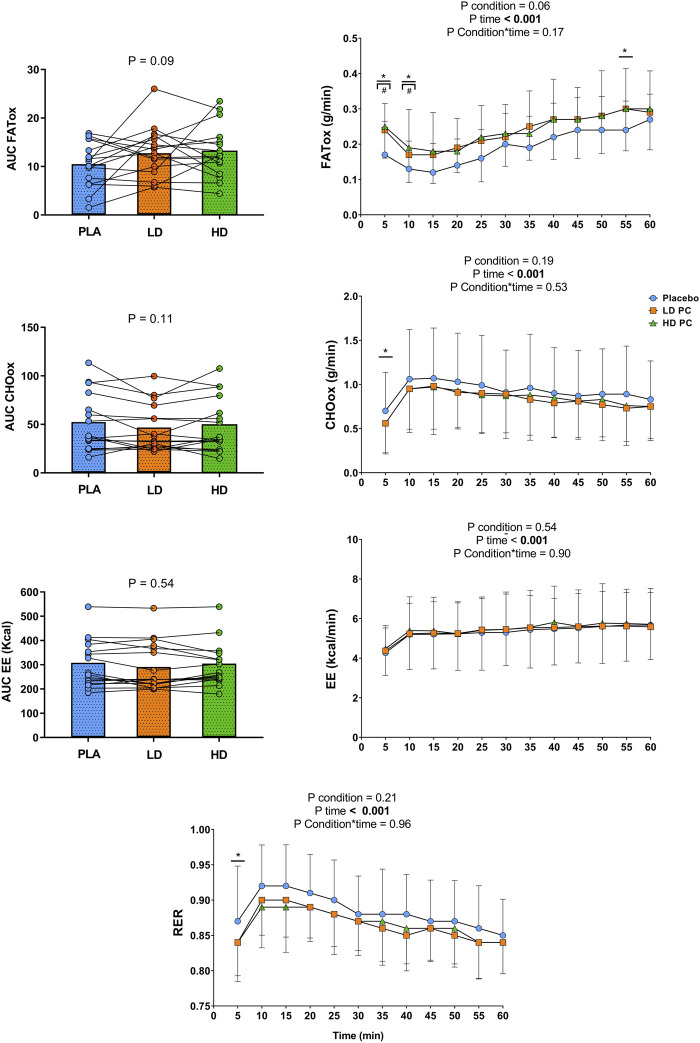
Two-way repeated measures analysis of variance (ANOVA) comparing the effects of phenylcapsaicin and placebo (PLA, HD, LD) across time on substrate oxidation, energy expenditure and respiratory exchange ratio during the 60 min steady-state test. AUC, Area Under the curve; FATox, Fat oxidation; CHOox, Carbohydrates oxidation; EE, Energy expenditure; RER, Respiratory exchange ratio; PLA, Placebo; HD, High dose; LD, Low dose; PC, Phenylcapsaicin. Intra-test analysis (one-way repeated measures ANOVA): *p_*ANOVA*_ ≤ 0.05; #*p*_*Bonferroni*_ ≤ 0.05.

No significant differences were found for AUC EE, AUC FATox, and AUC CHOox (*p* range = 0.09 to 0.54; ƞp^2^ range = 0.04–0.18). Two-way repeated measures ANOVAs exhibited non-significant differences for condition in FAToxidation (*p* = 0.06; ƞp^2^ = 0.14), CHOox (*p* = 0.19; ƞp^2^ = 0.10), EE (*p* = 0.54; ƞp^2^ = 0.008), and RER (*p* = 0.21; ƞp^2^ = 0.10). However, two-way repeated measures ANOVA revealed significant differences for time in all the aforementioned variables (*p* ≤ 0.001; ƞp^2^ range = 0.54–0.79) but not for condition × time interaction (*p* range = 0.17 to 0.96; ƞp^2^ range = 0.003–0.06). Intra-test one-way repeated measures ANOVA only exhibited a significant effect on FATox at min 5 (*p* = 0.005; ƞp^2^ = 0.28), 10 (*p* ≤ 0.001; ƞp^2^ = 0.29), and 55 (*p* = 0.04; ƞp^2^ = 0.24) for HD and LD and for CHOox at min 5 (*p* = 0.05; ƞp^2^ = 0.25) and RER (*p* = 0.003; ƞp^2^ = 0.25) at min 5 in favor of PLA but not for any variable in any other stage. Post-hoc Bonferroni reported significant differences in favor of HD and LD between PLA/HD at 5 min (*p* = 0.002; d = 0.92), PLA/LD at 5 min (*p* = 0.002; d = 0.74), PLA/HD (*p* = 0.002; d = 0.66) and PLA/LD at 10 min (*p* = 0.002; d = 0.56) for FATox (Figure 3).

### 3.5 Maximal effort test

None of the variables (i.e., heart rate, lactate, RPEg at 30% and 90%, and RPEquad) recorded during the maximal effort test differed between the experimental conditions (*p* ranged from 0.011 to 0.915) except RPEg at 60% due to the lower values of HD compared to LD and PLA (*p* = 0.05) (Table 4).

**TABLE 4:** One-way repeated measures analysis of variance (ANOVA) or Friedman’s test comparing different variables obtained during the incremental test.

Variable	Conditions			ANOVA or Friedman’s
	PLA	LD	HD	
Final stage (W)	296.4 ± 43.7	291.1 ± 43.4	292.9 ± 37.2	F = 0.48, *p* = 0.62, ƞp^2^ = 0.05
VO2_max_ (mL/min/kg)	30.9 ± 7.3	30.5 ± 7.4	30.9 ± 5.8	F = 0.08, *p* = 0.91, ƞp^2^ = 0.01
Lactate (mmol/L)	9.0 ± 3.7	8.0 ± 2.8	8.4 ± 3.3	F = 0.51, *p* = 0.60, ƞp^2^ = 0.04
RPEg 30% (a.u.)	1.4 ± 1.3	1.7 ± 1.2	1.4 ± 0.8	χ = 1.08, *p* = 0.58
RPEg 60% (a.u.)	3.8 ± 1.8	3.9 ± 2.0	3.4 ± 1.9	χ = 5.84, *p* = 0.05*
RPEg 90% (a.u.)	7.1 ± 1.6	7.0 ± 1.7	6.9 ± 1.7	χ = 0.62, *p* = 0.73
RPEquad 30% (a.u.)	1.8 ± 1.4	2.1 ± 1.4	1.8 ± 1.3	χ = 0.97, *p* = 0.61
RPEquad 60% (a.u.)	4.8 ± 1.5	4.9 ± 1.5	4.4 ± 1.5	χ = 3.73, *p* = 0.15
RPEquad 90% (a.u.)	7.9 ± 0.9	7.9 ± 0.9	8.1 ± 1.2	χ = 2.60, *p* = 0.27
Heart rate 30% (bpm)	106.5 ± 11.1	107.7 ± 9.4	105.8 ± 12.5	F = 0.44, *p* = 0.64, ƞp^2^ = 0.03
Heart rate 60% (bpm)	140.2 ± 15.6	139.9 ± 15.0	135.9 ± 13.8	F = 2.40, *p* = 0.11, ƞp^2^ = 0.15
Heart rate 90% (bpm)	169.9 ± 15.0	171.0 ± 13.7	170.1 ± 14.9	F = 0.48, *p* = 0.49, ƞp^2^ = 0.03

Mean ± standard deviation. ANOVA, analysis of variance; PLA, Placebo; LD, low dose; HD, high dose; VO2_max_, maximal oxygen consumption; RPEg, general rate of perceived exertion; RPEquad, quadriceps rate of perceived exertion; a.u., arbitrary units. * Significant difference: *p* ≤ 0.05.

## 4 Discussion

This study aimed to evaluate for the first time the effects of two doses of PC on several metabolic and perceptual responses during a steady-state and a maximal incremental test. The main finding of this study was that LD and HD of PC increase fat oxidation during different stages of the steady-state test and the peak of fat oxidation (FOpeak) in comparison to PLA. Furthermore, both PC doses, exhibited a reduction in CHOox and RER during the first stage of the steady-state test which suggest a shift on substrate oxidation. HD of PC also reduced maximum heart rate during the steady-state test. Contrary to our hypothesis, LD elicited higher RPEg values compared to PLA and HD during the steady-state test. Intriguingly, although skin body temperature was not affected by PC, the thermal perception was significantly lower in the supraclavicular area for HD. Nevertheless, only RPEg at 60% of maximal intensity was lowered with the HD of PC supplementation during the incremental test. Therefore, the present results suggest that PC enhances fat oxidation during aerobic exercise and modulate the perceptual responses to exercise and the maximum heart rate. However, these perceptual and heart rate effects are only reported when the HD is ingested.

In the present study, the FOpeak during the steady-state test and the rate of fat oxidation during different stages of this test were higher for HD and LD in comparison to PLA (see Figure 3). In addition, both PC doses also exhibited lower carbohydrates oxidation and RER values during the first stage of the steady-state test, which may suggest a shift on substrate oxidation induced by PC (de Moura e Silva et al., 2021). These results are reported for the first time with the use of PC. Furthermore, only two studies have previously documented the metabolic effects of a capsaicinoid on substrate oxidation, reporting contradictory findings between them (Lim et al., 1997; Rossi et al., 2022). The increase of fat oxidation reported in the current study might be explained by the exercise protocol employed and the type of supplement ingested (EFSA, 2012; Turck et al., 2019). Additionally, contrary to previous research, in the present study MFO and FATmax were calculated for each participant which matched the metabolic and mechanical training intensities of the subjects. This fact is essential due to different factors such as the exercise intensity, metabolic cart use, or the ergometer employed, influence the metabolic responses during exercise (Amaro-Gahete et al., 2018; Amaro-Gahete et al., 2019). Concerning the type of supplement used, previous studies revealed that capsinoids supplementation does not modulate the metabolic responses during aerobic exercise (Osuna-Prieto et al., 2022; Rossi et al., 2022). Although a MFO protocol was used by Osuna-Prieto et al. (2022), previous research has reported that capsinoids are hydrolyzed in the gastrointestinal tract, and their metabolites are excreted rapidly after their ingestion (Bernard et al., 2008). Capsiate circulating levels are significantly much lower in comparison to capsaicin levels, and their final linkage with TRPV1 is approximately 1/10 compared to capsaicinoids such as PC (Sasahara et al., 2010). Accordingly, PC might be able to activate in a higher way TRPV1 receptors leading to a greater increase in fatty acid oxidation and increasing glycogen sparing. This finding may be important due to the relevant link between muscle glycogen and performance in aerobic sports (Murray and Rosenbloom, 2018).

Although the TRPV1 temperature threshold is set at 43°C on dorsal root ganglia cells, capsaicin seems to decrease the activation threshold to 36.8°C (Szolcsányi, 2015). In humans, topical capsaicin has been demonstrated to increase heat loss by increasing peripheral vasodilation thereby improving the skin’s vasoconstrictive tone, increasing heat dissipation during exercise and increasing thermal perception and heat stress (Botonis et al., 2019). However, oral capsiate supplementation has not exhibited any of these effects on overweight participants (Osuna-Prieto et al., 2022). As our study presents an encapsulated formulation of PC, the null effect on skin body temperature may be related to the nature of the vehicle used. Accordingly, if a non-encapsulated powder formulation might have been ingested, a thermoregulatory response could be expected due to the direct contact of PC with the esophagus and gastrointestinal tissues (Szolcsányi, 2015). However, in the present study, the thermal perception was reduced in the clavicle area for the HD condition. Because PC was encapsulated and a burning reflux effect was not reported by any of the participants, this finding contrasts most literature where capsaicinoids spiciness is discussed (Naves et al., 2019). A plausible explanation for this issue may be that the digestion of the capsules did not produce an irritant effect, albeit they may have increase the internal temperature (i.e., not directly measured with skin temperature assessment) (Patcharatrakul et al., 2020). As a consequence, subjects may have experienced a counterregulatory response to PC at the end of the test on the aforementioned area (see Figure 2). Moreover, LD seems to be an insufficient dose to alter the termal perception. Besides, as heat perception contributes to the development of fatigue during exercise, this finding may be helpful to counteract fatigue in aerobic sports (Willmott et al., 2020). Overall, future research should assess if this finding depends on the ingestion of PC instead of traditional capsaicin formulations, the effect of the application vehicle on capsaicin’s pungency perception and the plausible existence of a counterregulatory mechanism of PC on thermal perception.

The effects of capsaicin and red peppers on cardiovascular responses are currently controversial due to the vast heterogeneity between protocols and doses used in previous literature (Shirani et al., 2021). However, recent research has shown that supplementation with purified capsaicin does not alter heart rate during an incremental exercise test in a cycloergometer until exhaustion (Giuriato et al., 2022). These studies employed intensities above 80% of VO2_max_ (Grgic et al., 2022), which therefore are not extrapolable to lower intensities. In the present study, although the medium heart rate did not differ between conditions, HD exhibited a significantly lower maximum heart rate during the steady-state test. As in the present study the intensity was matched in the steady-state test, it is possible that under HD supplementation, subjects experienced a higher mechanical efficiency, which may have reduced the relative workload, resulting in lower cardiovascular demands during the test (Mikus et al., 2009). In addition, circulating lactate response across time trended to be lower with both PC doses in the steady-state test. Previous preclinical research has shown that lactate is a potent endogenous inhibitor of TRPV1 activity (de la Roche et al., 2016). According to this possible mechanism, PC may have modulated sarcoplasmic calcium efflux channels lowering lactate levels during both tests (de la Roche et al., 2016). This finding agrees with previous research in resistance training and high-intensity running (de Freitas et al., 2018b; de Freitas et al., 2018a). Furthermore, this lactate lowering effect of PC may also be partially explained by the shift on susbtrate oxidation produced by this substance and provides novel information about the physiological independence between this effect and the intensity of the exercise used. However, contrary to previous research where capsaicin supplementation led to lower RPE values, in our data, participants showed higher RPE scores under the LD condition, and improvements were not reported with HD compared to PLA (de Freitas et al., 2018a). The low intensity demanded in this task may be insufficient to report an ergogenic effect of PC on perceptual variables (Jiménez Martínez et al., 2023). However, LD of PC reported for the first time a worsening effect of a capsaicinoid on RPE. Therefore, if PC at LD in low intensity tasks produces a counterregulatory effect on perceptual performance or if the sensitivity of RPE under these novel conditions is altered, should be corroborate in further studies.

Concerning the incremental test, the HD of PC reduced RPEg at 60% of the maximal intensity achieved. Additionally, the reduction in RPE values during high-intensity exercise after capsaicin supplementation is well documented (Jiménez-Martínez et al., 2022). During high-intensity exercise capsaicinoids reduce perceived exertion due to TRPV1 activation. TRPV1 are linked to afferent III and IV nerve fibers (Collins et al., 2018). These fibers influence central fatigue during exercise, which finally reduce motorneuron firing during high-intensity tasks (Alix-Fages et al., 2022b; Jiménez Martínez et al., 2023). For this reason, HD might have produce a “desensitizer” effect leading to a lower percepetion of exertion in the maximal incremental test. This threshold may not be achieved by the LD. Additionally, these findings align with previous research that has reported that capsaicin supplementation does not increase VO2_max_ or any other metabolic outcome during high-intensity exercise, although the time to exhaustion in interval training is increased (de Freitas et al., 2019).

## 5 Strengths and limitations

This study presents essential strengths such as the control of the fasting conditions and the meal before each session, and the randomized, triple-blinded, crossover design. Nonetheless, some limitations should be addressed. First, the study only included active males, and these results may not be extrapolated to other populations. Secondly, these results can not be extrapolated to other exercise modalities or intensities. Finally, the chronic effects of PC on metabolic responses during exercise should not be extrapolated from this acute study.

## 6 Conclusion

The results of the present study suggest that LD and HD of PC modulate the metabolic response (FATox, CHOox and RER) to exercise and HD of PC reduces maximum heart rate values during aerobic exercise. However, PC only improves the perceptual responses (i.e., RPEg and clavicle thermal perception) to exercise when it is consumed in HD.

## Data Availability

The raw data supporting the conclusion of this article will be made available by the authors, without undue reservation.
